# The within-person relationship of school climate and mental wellbeing in adolescence

**DOI:** 10.1093/eurpub/ckac129.616

**Published:** 2022-10-25

**Authors:** H Bjørnøy Urke, SM Kristensen, E Årdal, N Wiium, T Bøe, M Gaspar deMatos, T Larsen

**Affiliations:** 1 Department of Health Promotion and Development, University of Bergen, Bergen, Norway; 2 Department of Psychosocial Science, University of Bergen, Bergen, Norway; 3 Faculdade de Motricidade Humana, University of Lisbon, Lisbon, Portugal

## Abstract

**Background:**

School climate is recognized as important for adolescent mental wellbeing. However, there is a lack of longitudinal studies, and most research focus on school climate as an antecedent of mental wellbeing while the relation could be in the opposite direction or bidirectional. Further, no study has examined this relation with analyses that separate between- from within-person variability to properly assess intraindividual developmental processes.

**Methods:**

Using a random-intercept cross-lagged panel model we investigated the longitudinal reciprocal relations of perceptions of a caring school climate and mental wellbeing across three time points (T1, T2, T3) in adolescence, and if socioeconomic position (SEP) predicted perceptions of a caring school climate and mental wellbeing at each time point. The sample consisted of 1508 Norwegian adolescents (60.7% female; baseline mean age = 16.33, SD = .62).

**Results:**

Results showed positive cross-lagged effects at the within-person level from mental wellbeing to later perceptions of a caring school climate across all time points (T1->T2 b=.12* [.01, .22], T2->T3 b=.12* [.01, .23]), but no similar effects in the opposite direction. Positive concurrent effects from SEP were observed at one time point for mental wellbeing (T1 b=.10* [.01, .19]), and all time points for perceptions of a caring school climate (T1 b=.12* [.02, .22], T2 b=.11* [.02, .20], T3 b=.12* [.02, .22]), indicating SEP to be related to intraindividual fluctuations mainly in perceptions of a caring school climate.

**Conclusions:**

The findings support a unidirectional temporal relationship from mental wellbeing to perceptions of a caring school climate and underscore the importance of investigating the subject longitudinally and as a function of within-person fluctuations. From a public health view, the findings support the importance of systematic efforts to promote mental wellbeing to facilitate positive school experiences of adolescents of all social classes.

**Key messages:**

• The study found a unidirectional temporal relation at the within-person level in adolescents where mental wellbeing predicted perceptions of a caring school climate, but not the other way around.

• The findings underline the importance of systematic public health efforts to promote mental wellbeing as one avenue to facilitate positive school experiences of adolescents of all social.

